# Racial and ethnic differences in epithelial ovarian cancer risk: an analysis from the Ovarian Cancer Association Consortium

**DOI:** 10.1093/aje/kwae076

**Published:** 2024-05-21

**Authors:** Nicola S Meagher, Kami K White, Lynne R Wilkens, Elisa V Bandera, Andrew Berchuck, Michael E Carney, Daniel W Cramer, Kara L Cushing-Haugen, Susan Jordan, Scott H Kaufmann, Nhu D Le, Malcolm C Pike, Marjorie Riggan, Bo Qin, Joseph H Rothstein, Linda Titus, Stacey J Winham, Hoda Anton-Culver, Jennifer A Doherty, Ellen L Goode, Celeste Leigh Pearce, Harvey A Risch, Penelope M Webb, Linda S Cook, Marc T Goodman, Holly R Harris, Loic Le Marchand, Valerie McGuire, Paul D P Pharoah, Danja Sarink, Joellen M Schildkraut, Weiva Sieh, Kathryn L Terry, Pamela J Thompson, Alice S Whittemore, Anna H Wu, Lauren C Peres, Melissa A Merritt

**Affiliations:** Department of Population and Public Health Sciences, Keck School of Medicine, University of Southern California Norris Comprehensive Cancer Center, Los Angeles, CA, United States

**Keywords:** ovarian cancer, risk factors, race, ethnicity

## Abstract

Limited estimates exist on risk factors for epithelial ovarian cancer (EOC) in Asian, Hispanic, and Native Hawaiian/Pacific Islander women. Participants in this study included 1734 Asian (*n* = 785 case and 949 control participants), 266 Native Hawaiian/Pacific Islander (*n* = 99 case and 167 control participants), 1149 Hispanic (*n* = 505 case and 644 control participants), and 24 189 White (*n* = 9981 case and 14 208 control participants) from 11 studies in the Ovarian Cancer Association Consortium. Logistic regression models estimated odds ratios (ORs) and 95% CIs for risk associations by race and ethnicity. Heterogeneity in EOC risk associations by race and ethnicity (*P* ≤ .02) was observed for oral contraceptive (OC) use, parity, tubal ligation, and smoking. We observed inverse associations with EOC risk for OC use and parity across all groups; associations were strongest in Native Hawaiian/Pacific Islander and Asian women. The inverse association for tubal ligation with risk was most pronounced for Native Hawaiian/Pacific Islander participants (odds ratio (OR) = 0.25; 95% CI, 0.13-0.48) compared with Asian and White participants (OR = 0.68 [95% CI, 0.51-0.90] and OR = 0.78 [95% CI, 0.73-0.85], respectively). Differences in EOC risk factor associations were observed across racial and ethnic groups, which could be due, in part, to varying prevalence of EOC histotypes. Inclusion of greater diversity in future studies is essential to inform prevention strategies.

**This article is part of a Special Collection on Gynecological Cancers**.

## Introduction

Variation in the age-standardized incidence rates of ovarian cancer exists between racial and ethnic groups in the United States; Surveillance, Epidemiology, and End Results data (*International Classification of Diseases, Tenth Revision*, code C56, ovary only) show the highest incidence per 100 000 people in non-Hispanic White (hereafter, “White”; 10.5), Hispanic (10.4), and non-Hispanic Black (8.8) populations.^1^ Women of Asian ancestry (hereafter referred to as Asian), along with Native Hawaiian and Pacific Islander groups, are commonly aggregated in surveillance data and epidemiologic studies, with a combined incidence rate in Surveillance, Epidemiology, and End Results of 9.8 per 100 000.^1^ In the Hawaii Tumor Registry (2012-2016), in which cancer incidence rates were reported separately for Asian and Native Hawaiian groups, ovarian cancer incidence was highest in the White population (15.7), moderate in Native Hawaiian women (8.4), and lower in Japanese and Chinese American women (7.1 and 7.3, respectively).^2^

Studies of epithelial ovarian cancer (EOC) risk factor associations across racial and ethnic groups may provide important information to understand observed differences in incidence rates. Several reproductive and hormone-related factors are established risk factors for EOC; for example, postmenopausal hormone use and high body mass index (BMI) are positively associated with risk, whereas parity, oral contraceptive (OC) use, and tubal ligation are inversely associated with risk of EOC.^3^^-^^6^ Several studies have examined EOC risk factors across different racial and ethnic groups^7^^-^^13^; however, few included Asian and Native Hawaiian/Pacific Islander participants, and these groups are commonly reported in aggregate. The exception is the prospective Multiethnic Cohort Study, with 155 incident EOC cases in White, 93 in Black, 57 in Native Hawaiian, 161 in Japanese American, and 141 in Hispanic women that were identified during a median of 20 years of follow-up.^10^ Although no statistically significant interactions for risk factor associations by racial and ethnic group were observed in this study, inverse associations between parity and OC use with EOC risk were strongest among Japanese American women, and age at natural menopause and postmenopausal hormone use were associated with increased EOC risk only in Hispanic women. None of the investigated risk factors had a statistically significant association with EOC risk in Native Hawaiian women, although this may reflect the limited sample size.

The largest study to date^9^ comparing race- and ethnicity-specific risk associations for EOC used pooled data from the African American Cancer Epidemiology Study^11^ and 11 Ovarian Cancer Association Consortium (OCAC) studies based in the United States, Australia, and Canada. This earlier study^9^ showed generally similar directions of risk factor associations for EOC across racial and ethnic groups, and only hysterectomy showed heterogeneity between groups. Another recent OCAC study highlighted the complexity of hysterectomy as an exposure in relation to menopausal hormone therapy use and endometriosis.^14^ The present study is an extension of that prior OCAC analysis^9^; compared with the earlier study, 9 of the OCAC studies were included in both analyses, whereas 2 studies (the Connecticut Ovarian Cancer Study and the Hormones and Ovarian Cancer Prediction Study) were not included in the present study because the inclusion criteria (minimum of 10 Asian, Native Hawaiian/Pacific Islander, or Hispanic cases) were not met. This report included 2 additional case-control studies (the Mayo Clinic Ovarian Cancer Case-Control Study [MAY], and the New Jersey Ovarian Cancer Study [NJO]) as well as additional participants from the other studies. The novelty of the present study includes the addition of exposures (BMI, calculated as as weight (kg) divided by height (m^2^), at age 18 years; smoking; separation of first-degree family history into breast and/or ovarian cancer). Importantly, to our knowledge, this was the first consortium-based analysis of EOC risk factors to address our key aim of disaggregating Asian and Native Hawaiian/Pacific Islander participants, in part because of differences in risk factor profiles and incidence rates of EOC.

## Methods

### Study sample

Data were included from 11 OCAC case-control studies (*n* = 9 from the United States, 1 study from Australia, and 1 from Canada). Studies were eligible if they collected extensive epidemiologic risk factor data and had 10 or more cases with a self-reported Asian/Native Hawaiian/Pacific Islander or Hispanic race or ethnicity (Table 1). Racial and ethnic groups were preferentially derived from self-report on questionnaires using data from the OCAC core database and included a combined Asian/Native Hawaiian/Pacific Islander group, Black, Hispanic, and White. To subdivide Asian and Native Hawaiian/Pacific Islander participants from the combined group, expanded race data from self-report on questionnaires were provided from 5 studies that had the largest representation of these women: Diseases of the Ovary and their Evaluation Study (DOV); Hawaii Ovarian Cancer Case-Control Study (HAW); Ovarian Cancer in Alberta and British Columbia Study (OVA); Genetic Epidemiology of Ovarian Cancer (STA); Los Angeles County Case-Control Studies of Ovarian Cancer (USC). Given the location of studies from which participants were drawn, Asian participants represented those residing outside of Asia. When self-reported data were not available, genetic ancestry data (available for 173 of 377 women) were used to classify 167 Asian and 6 Native Hawaiian/Pacific Islander participants. Genetic ancestry was determined based on clusters created from the Oncoarray principal component analysis and/or the Collaborative Oncological Gene–Environment Study (COGS) principal component analysis; 85 participants had Oncoarray and COGS values (all concordant) and 88 participants had either Oncoarray or COGS data.^15^^,^^16^

**Table 1:** Participant numbers in each study by racial and ethnic group

			**Asian** ^**a**^		**Native Hawaiian/Pacific Islander** ^**a**^		**Hispanic**		**White, non-Hispanic**	
**Study acronym**	**Country**	**Dates of interview**	**Cases, no. (%)**	**Controls, no. (%)**	**Cases, no. (%)**	**Controls, no. (%)**	**Cases, no. (%)**	**Controls, no. (%)**	**Cases, no. (%)**	**Controls, no. (%)**
			785	949	99	167	505	644	9981	14 208
AUS	Australia	2002-2005	36 (4.6)	15 (1.6)	3 (3.0)	0	0	0	1487 (14.9)	1414 (10.0)
DOV	United States	2002-2009	58 (7.4)	54 (5.7)	6 (6.1)	8 (4.8)	35 (6.9)	42 (6.5)	1016 (10.2)	1679 (11.8)
HAW	United States	1993-2008	314 (40.0)	509 (53.6)	86 (86.9)	156 (93.4)	32 (6.3)	36 (5.6)	267 (2.7)	385 (2.7)
MAY	United States	2000-2008	4 (0.5)	2 (0.2)	0	0	9 (1.8)	11 (1.7)	1582 (15.9)	2249 (15.8)
NCO	United States	1999-2008	4 (0.5)	3 (0.3)	1 (1.0)	0	7 (1.4)	12 (1.9)	814 (8.2)	856 (6.0)
NEC	United States	1992-2008	12 (1.5)	8 (0.8)	0	0	18 (3.6)	33 (5.1)	1429 (14.3)	2031 (14.3)
NJO	United States	2002-2008	2 (0.3)	0	0	0	12 (2.4)	23 (3.6)	207 (2.1)	399 (2.8)
OVA	Canada	2002-2012	97 (12.4)	118 (12.5)	0	0	6 (1.2)	20 (3.1)	1133 (11.4)	2373 (16.7)
STA	United States	1997-2002	82 (10.5)	77 (8.1)	2 (2.0)	3 (1.8)	51 (10.1)	62 (9.6)	327 (3.3)	349 (2.5)
UCI	United States	1995-2005	16 (2.0)	12 (1.3)	0	0	36 (7.1)	39 (6.1)	369 (3.7)	534 (3.8)
USC	United States	1993-2010	160 (20.4)	151 (15.9)	1 (1.0)	0	299 (59.2)	366 (56.8)	1350 (13.5)	1939 (13.7)

Abbreviations: AUS, Australian Ovarian Cancer and Australian Cancer Study; BMI, body mass index; DOV, Diseases of the Ovary and their Evaluation Study; HAW, Hawaii Ovarian Cancer Case-Control Study; MAY, Mayo Clinic Ovarian Cancer Case-Control Study; NCO, North Carolina Ovarian Cancer Study; NEC, New England Case-Control Study of Ovarian Cancer; NJO, New Jersey Ovarian Cancer Study; OC, oral contraceptive; OR, odds ratio; OVA, Ovarian Cancer in Alberta and British Columbia Study; STA, Genetic Epidemiology of Ovarian Cancer; UCI, University of California, Irvine, Ovarian Cancer Study; USC, Los Angeles County Case-Control Studies of Ovarian Cancer.

^a^ Asian includes Chinese, Japanese, Korean, Filipino, Vietnamese, Thai. Native Hawaiian/Pacific Islander includes Hawaiian, Pacific Islander (Tongan, Samoan, Māori, Palauan, Chuukese, Micronesian).

Exposures of interest were assessed up to the reference date (diagnosis date for cases, interview date for control participants), unless otherwise specified. Exposures were categorized as follows: age (18-29, 30-39, 40-49, 50-59, 60-69, ≥70 years); age at menarche (<12, 12-13, ≥14 years); duration of OC use (never, < 5, ≥5 years); parity (0, 1, 2, ≥3 live births); tubal ligation at least 1 year prior to the reference date (no, yes); breastfeeding (no, yes); menopausal status (pre-/peri- or postmenopausal); and postmenopausal hormone use (no, yes; use of estrogen only, estrogen plus progesterone, and unknown formulation types); endometriosis (no, yes); hysterectomy at least 1 year prior to the reference date (no, yes); recent BMI, defined as 1 year prior to reference date for all study sites except for DOV and HAW, where BMI was self-reported 5 years prior to the reference date (<18.5, 18.5-24.9, 25.0-29.9, 30-34.9, ≥35.0 kg/m^2^; and a binary comparison of ≥30 vs 18.5-24.9); BMI at age 18 years (<18.5, 18.5-24.9, 25.0-29.9, ≥30); smoking (never, former, current); first-degree family history of breast cancer (no, yes); and first-degree family history of ovarian cancer (no, yes). First-degree family history refers to the mother, sister, and/or daughter.

Eligible participants were aged 18-99 years at the reference date. Eligible cases were women diagnosed with invasive EOC (including fallopian tube and primary peritoneal cancers) and control participants were women who had no previous history of ovarian cancer and at least 1 intact ovary at study recruitment. In this analysis, a total of 29 257 participants from the 11 studies were eligible for inclusion. Participants were excluded if they were not in the 4 racial and ethnic groups that were the focus of this study (Asian, Hispanic, Native Hawaiian/Pacific Islander, and White; *n* = 1715) or data were not available to disaggregate Asian and Native Hawaiian/Pacific Islander participants (*n* = 204), leaving 27 338 participants for the present study (*n* = 11 370 case and 15 968 control participants). Our present analysis did not include Black women because an earlier study had been published using a much larger sample size of these women,^9^ facilitated by pooling data from the OCAC studies with the African American Cancer Epidemiology Study.

Participants in each study provided informed consent and all studies have institutional review board/human research ethics committee approval for the work presented.

### Statistical analysis

Multivariable logistic regression models were used to estimate odds ratios (ORs) and 95% CIs for the association of each exposure variable (all variables previously listed, except for age and menopausal status) and risk of EOC across 4 racial and ethnic groups: Asian, Hispanic, Native Hawaiian/Pacific Islander, and White. All models included study site, age group, and racial and ethnic group as strata variables, the exposure variable, and interaction terms for racial and ethnic group, and the exposure variable. Oral contraceptive use and parity (including a missing data category) were also included a priori as adjustment variables (when not an exposure variable). Models for postmenopausal hormone use and breastfeeding were based on postmenopausal women and parous women only, respectively. Tests for linear trend (*P* for trend) were performed using variables that were based on the median values for each category, when applicable. Heterogeneity across racial and ethnic groups for each exposure was assessed by calculating the *P* value for heterogeneity using the global Wald test on the interaction terms (race and ethnicity and categorical exposure variable(s); or race and ethnicity and trend exposure variable). Analyses were repeated but limited to high-grade serous tubo-ovarian carcinoma (HGSC; *n* = 7233) cases compared with all control participants, where HGSC was defined as serous histology grades 2-4 (*n* = 5496); serous histology with missing/unknown grade (*n* = 1146); endometrioid histology grades 3-4 (*n* = 446) (to avoid misclassification of HGSC as endometroid^17^); or poorly differentiated epithelial histology grades 2-4 (*n* = 145). Heterogeneity in risk associations between study sites was assessed using the SAS metaanal macro^18^ and was restricted to White participants only due to the sparsity of racial and ethnic groups within individual studies. Age-standardization to the age distribution of the study population using 10-year age groups was performed for descriptive analysis of control participants. All analyses were performed using SAS, version 9.4.

## Results

We noted variation in the prevalence of different risk factors by racial and ethnic group (Table 2). Asian, Hispanic, and Native Hawaiian/Pacific Islander control participants were younger (43%-49% of control participants were aged < 50 years) compared with White participants (27% of control participants were aged < 50 years). A high proportion of Native Hawaiian/Pacific Islander control participants had a recent BMI ≥30 (40%) compared with Asian (6%), White (20%), and Hispanic (24%) control groups. A higher percentage of Native Hawaiian/Pacific Islander control participants, compared with other racial or ethnic groups, reported an earlier age at menarche (<12 years; 34% and ≤ 26%, respectively) and a tubal ligation (42% and ≤ 26%, respectively). A higher proportion of Native Hawaiian/Pacific Islander, compared with Asian, White, and Hispanic control participants, reported ≥3 live births (60% compared to 34%, 35%, and 46%, respectively). Endometriosis was reported by a slightly higher proportion of White (8%) and Asian (7%) control participants compared with ≤ 6% from the other racial and ethnic groups. Native Hawaiian/Pacific Islander control participants more frequently reported a family history of breast cancer (40%) compared with ≤ 17% from other racial and ethnic groups. Reports of a family history of ovarian cancer were highest in Hispanic (6%) control participants, lowest in Native Hawaiian/Pacific Islander control participants (1%), and frequencies of 4% and 3% were reported in Asian and White control participants, respectively. For all results presented in Table 2, we observed similar findings after age standardization to facilitate comparisons across control participants (Table S1).

**Table 2:** Participant characteristics by racial and ethnic group^a^

Variable	**Asian^**b**^**		**Native Hawaiian/Pacific Islander** ^**b**^		**Hispanic**		**White, non-Hispanic**	
	**Cases, no. (%)**	**Controls, no. (%)**	**Cases, no. (%)**	**Controls, no. (%)**	**Cases, no. (%)**	**Controls, no. (%)**	**Cases, no. (%)**	**Controls, no. (%)**
Total no.	785	949	99	167	505	644	9981	14 208
Age at reference, y^c^								
18-29	13 (1.7)	44 (4.6)	2 (2.0)	19 (11.4)	7 (1.4)	45 (7.0)	79 (0.8)	256 (1.8)
30-39	86 (10.9)	107 (11.3)	14 (14.1)	22 (13.2)	42 (8.3)	84 (13.0)	406 (4.1)	889 (6.3)
40-49	223 (28.4)	256 (27.0)	18 (18.2)	39 (23.4)	123 (24.4)	187 (29.0)	1611 (16.1)	2701 (19.0)
50-59	218 (27.8)	261 (27.5)	36 (36.4)	37 (22.2))	166 (32.9)	181 (28.1)	3150 (31.6)	4341 (30.6)
60-69	145 (18.5)	146 (15.4)	21 (21.2)	35 (21.0)	115 (22.8)	113 (17.6)	2969 (29.8)	3859 (27.2)
≥70	100 (12.7)	135 (14.2)	8 (8.1)	15 (9.0)	52 (10.3)	34 (5.3)	1766 (17.7)	2162 (15.2)
Age at menarche, y								
<12	144 (18.6)	207 (21.9)	28 (28.6)	57 (34.1)	119 (23.8)	163 (25.7)	1908 (20.0)	2702 (19.4)
12-13	363 (47.0)	440 (46.6)	48 (49.0)	75 (44.9)	255 (50.9)	301 (47.5)	5267 (55.3)	7653 (55.0)
≥14	266 (34.4)	297 (31.5)	22 (22.5)	35 (21.0)	127 (25.4)	170 (26.8)	2358 (24.7)	3566 (25.6)
Missing data	12	5	1	0	4	10	448	287
Education								
<High school	100 (13.1)	83 (9.0)	17 (17.2)	9 (5.4)	136 (34.3)	129 (25.5)	856 (9.3)	781 (5.9)
≥High school	663 (86.9)	837 (91.0)	82 (82.8)	158 (94.6)	260 (65.7)	376 (74.5)	8334 (90.7)	12487 (94.1)
Missing data	22	29	0	0	109	139	791	940
OC use								
Never	523 (67.1)	454 (48.0)	59 (59.6)	54 (32.3)	258 (51.5)	258 (40.8)	3746 (39.0)	3765 (27.0)
<5 y	185 (23.8)	300 (31.7)	29 (29.3)	82 (49.1)	165 (32.9)	222 (35.1)	3261 (33.9)	4557 (32.7)
≥5 y	71 (9.1)	192 (20.3)	11 (11.1)	31 (18.6)	78 (15.6)	153 (24.2)	2607 (27.1)	5631 (40.4)
Missing data	6	3	0	0	4	11	367	255
Parity								
0 live births	266 (34.1)	176 (18.6)	27 (27.6)	17 (10.2)	98 (19.5)	93 (14.7)	2360 (24.3)	2388 (17.0)
1	133 (17.0)	153 (16.1)	13 (13.3)	19 (11.4)	72 (14.3)	85 (13.4)	1323 (13.6)	1801 (12.8)
2	186 (23.8)	300 (31.6)	25 (25.5)	30 (18.0)	116 (23.1)	165 (26.0)	2925 (30.1)	4898 (34.8)
≥3	196 (25.1)	320 (33.7)	33 (33.7)	101 (60.5)	216 (43.0)	292 (46.0)	3122 (32.1)	4993 (35.5)
Missing data	4	0	1	0	3	9	251	128
Tubal ligation								
No	683 (87.8)	733 (78.1)	85 (85.9)	97 (58.1)	408 (82.4)	462 (74.3)	7130 (81.5)	8855 (75.9)
Yes	95 (12.2)	206 (21.9)	14 (14.1)	70 (41.9)	87 (17.6)	160 (25.7)	1624 (18.6)	2815 (24.1)
Missing data	7	10	0	0	10	22	1227	2538
Breastfeeding^d^								
No	136 (27.2)	167 (22.1)	22 (31.9)	38 (25.3)	113 (35.8)	135 (32.5)	2294 (39.0)	2802 (30.4)
Yes	364 (72.8)	590 (77.9)	47 (68.1)	112 (74.7)	203 (64.2)	281 (67.6)	3596 (61.1)	6416 (69.6)
Missing data	15	16	2	0	88	126	1480	2474
Menopausal status								
Pre/perimenopause	333 (42.9)	455 (48.6)	33 (33.7)	77 (46.4)	174 (34.9)	320 (50.2)	2506 (25.8)	4392 (31.5)
Postmenopause	444 (57.1)	481 (51.4)	65 (66.3)	89 (53.6)	325 (65.1)	318 (49.8)	7178 (74.1)	9541 (68.5)
Missing data	8	13	1	1	6	6	297	275
Postmenopausal hormone use^e^								
No	272 (61.4)	262 (54.5)	51 (78.5)	58 (65.2)	206 (63.4)	195 (61.7)	3566 (51.0)	4576 (48.4)
Yes	171 (38.6)	219 (45.5)	14 (21.5)	31 (34.8)	119 (36.6)	121 (38.3)	3423 (49.0)	4879 (51.6)
Missing data	1	0	0	0	0	4	189	86
Endometriosis								
No	524 (87.6)	696 (92.7)	90 (93.8)	155 (94.5)	412 (93.4)	526 (95.6)	7161 (89.2)	10416 (92.3)
Yes	74 (12.4)	55 (7.3)	6 (6.3)	9 (5.5)	29 (6.6)	24 (4.4)	864 (10.8)	868 (7.7)
Missing data	187	198	3	3	64	94	1956	2924
Hysterectomy								
No	699 (90.2)	876 (92.5)	90 (90.9)	151 (90.4)	395 (82.10)	555 (89.1)	6473 (80.0)	9881 (82.9)
Yes	76 (9.8)	71 (7.5)	9 (9.1)	16 (9.6)	86 (17.9)	68 (10.9)	1619 (20.0)	2033 (17.1)
Missing data	6	0	0	0	15	10	307	45
BMI, recent^f^								
<18.5 kg/m^2^	31 (5.2)	28 (3.8)	0	2 (1.2)	4 (0.9)	4 (0.7)	170 (2.1)	235 (2.1)
18.5-24.9	366 (61.7)	495 (66.4)	27 (28.4)	53 (32.3)	163 (37.1)	231 (42.2)	3634 (44.9)	5473 (48.5)
25.0-29.9	140 (23.6)	177 (23.7)	31 (32.6)	44 (26.8)	142 (32.4)	179 (32.7)	2351 (29.1)	3267 (29.0)
30.0-34.9	38 (6.4)	38 (5.1)	17 (17.9)	27 (16.5)	70 (16.0)	71 (13.0)	1118 (13.8)	1419 (12.6)
≥35.0	18 (3.0)	8 (1.1)	20 (21.1)	38 (23.2)	60 (13.7)	63 (11.5)	819 (10.1)	883 (7.8)
Missing data	192	203	4	3	66	96	1889	2931

**(Table continues)**

**Table 2:** Continued.

Variable	**Asian^**b**^**		**Native Hawaiian/Pacific Islander** ^**b**^		**Hispanic**		**White, non-Hispanic**	
	**Cases, no. (%)**	**Controls, no. (%)**	**Cases, no. (%)**	**Controls, no. (%)**	**Cases, no. (%)**	**Controls, no. (%)**	**Cases, no. (%)**	**Controls, no. (%)**
BMI, age 18 y								
<18.5	133 (23.2)	141 (19.4)	10 (10.3)	9 (5.4)	56 (13.2)	73 (13.6)	1110 (16.6)	1634 (18.0)
18.5-24.9	402 (70.0)	540 (74.4)	60 (61.9)	106 (64.6)	303 (71.5)	394 (73.6)	4938 (73.6)	6754 (74.3)
25.0-29.9	30 (5.2)	40 (5.5)	15 (15.5)	31 (18.9)	49 (11.6)	52 (9.7)	512 (7.6)	547 (6.0)
≥30.0	9 (1.6)	5 (0.7)	12 (12.4)	18 (11.0)	16 (3.8)	16 (3.0)	148 (2.2)	161 (1.8)
Missing data	211	223	2	3	81	109	3273	5112
Smoking								
Never	636 (83.1)	688 (74.2)	58 (58.6)	86 (51.5)	248 (62.3)	323 (63.7)	4959 (53.3)	7168 (53.1)
Former	32 (4.2)	87 (9.4)	14 (14.1)	41 (24.6)	107 (26.9)	132 (26.0)	3192 (34.3)	4858 (36.0)
Current	97 (12.7)	152 (16.4)	27 (27.3)	40 (24.0)	43 (10.8)	52 (10.3)	1162 (12.5)	1464 (10.9)
Missing data	20	22	0	0	107	137	668	718
Family history of breast cancer								
No	466 (87.1)	476 (84.3)	39 (69.6)	34 (59.7)	324 (83.9)	425 (89.9)	6649 (79.8)	10259 (83.2)
Yes	69 (12.9)	89 (15.8)	17 (30.4)	23 (40.4)	62 (12.1)	48 (10.2)	1679 (20.2)	2073 (16.8)
Missing data	250	384	43	110	119	171	1653	1876
Family history of ovarian cancer								
No	489 (95.7)	511 (96.2)	47 (94.0)	40 (97.6)	346 (91.8)	436 (94.4)	7656 (93.5)	11792 (97.0)
Yes	22 (4.3)	20 (3.8)	3 (6.0)	1 (1.1)	31 (8.2)	26 (5.6)	530 (6.5)	366 (3.0)
Missing data	274	418	49	126	128	182	1795	2050
Histology/grade								
High-grade serous	352 (44.8)		46 (46.5)		323 (64.0)		6512 (65.2)	
Low-grade serous	5 (0.6)		0		26 (5.1)		310 (3.1)	
Endometrioid	98 (12.5)		13 (13.1)		48 (9.5)		999 (10.0)	
Mucinous	85 (10.8)		13 (13.1)		42 (8.3)		482 (4.8)	
Clear cell	148 (18.9)		15 (15.2)		24 (4.8)		675 (6.8)	
Mixed cell	16 (2.0)		1 (1.0)		4 (0.8)		293 (2.9)	
Other epithelial	81 (10.4)		11 (11.1)		38 (7.5)		710 (7.1)	
Stage								
Localized	221 (28.2)		34 (34.3)		98 (19.1)		1416 (14.2)	
Regional	146 (18.6)		20 (20.2)		75 (14.6)		1397 (14.0)	
Distant	312 (39.8)		43 (43.4)		314 (61.2)		5912 (59.3)	
Unknown stage	106 (13.5)		2 (2.0)		18 (3.6)		1256 (12.6)	

Abbreviations: BMI, body mass index; DOV, Diseases of the Ovary and their Evaluation Study; HAW, Hawaii Ovarian Cancer Case-Control Study; MAY, Mayo Clinic Ovarian Cancer Case-Control Study; NJO, the New Jersey Ovarian Cancer Study; OC, oral contraceptive; OVA, Ovarian Cancer in Alberta and British Columbia Study; STA, Genetic Epidemiology of Ovarian Cancer; USC, Los Angeles County Case-Control Studies of Ovarian Cancer.

^a^ The following variables were missing for certain study sites: breastfeeding (MAY); endometriosis (OVA, STA); BMI recent (OVA, STA); BMI at age 18 (MAY, OVA, STA); postmenopausal hormone use (STA). One study (MAY) was an outlier and was excluded from hysterectomy analysis.

^b^ Asian includes Chinese, Japanese, Korean, Filipino, Vietnamese, Thai. Native Hawaiian/Pacific Islander includes Hawaiian, Pacific Islander (Tongan, Samoan, Māori, Palauan, Chuukese, Micronesian).

^c^ Reference date is defined as age at diagnosis for cases and age at interview for control participants.

^d^ Breastfeeding refers to parous women only.

^e^Postmenopausal hormone use refers to use of estrogen only, estrogen plus progesterone, and unknown formulation types among postmenopausal women only.

^f^ Recent BMI refers to 1 year before the reference date for all sites, except for DOV and HAW (5 years before the reference date). BMI calculated as weight (kg) divided by height (m^2^).

Among EOC cases, Hispanic and White women were more commonly diagnosed with HGSC (64% and 65% of all EOC, respectively, compared with 45% in Asian and 46% in Native Hawaiian/Pacific Islander EOC cases) (Table 2). Clear cell carcinoma was observed more frequently among Asian and Native Hawaiian/Pacific Islander women (19% and 15%, respectively) and in ≤ 7% of Hispanic and White cases. Mucinous carcinoma was slightly more prevalent in Native Hawaiian/Pacific Islander and Asian cases (13% and 11%, respectively), compared with 8% of Hispanic and 5% of White cases. Hispanic and White patients were most often diagnosed with a distant stage of disease (61% and 60%, respectively); however, this was observed in less than half of Native Hawaiian/Pacific Islander (43%), and Asian (40%) cases.

In our analysis of factors that were expected to lower the risk of EOC, we observed significant heterogeneity in risk associations across racial and ethnic groups for increasing duration of OC use, parity, and tubal ligation (*P* ≤ .0001 for heterogeneity) (Table 3). An inverse association between increasing duration of OC use and EOC risk was present within each racial and ethnic group (*P* ≤ .002 for trend), with the most pronounced risk reduction observed for Asian participants (≥5 years of OC use vs never use, OR = 0.31; 95% CI, 0.22-0.42) and Native Hawaiian/Pacific Islander participants (≥5 years use vs never use, OR = 0.33; 95% CI, 0.14-0.79), followed by White and Hispanic participants. Associations with parity were observed for all racial and ethnic groups and were strongest among Native Hawaiian/Pacific Islander women (≥3 vs no live births, OR = 0.10; 95% CI, 0.04-0.25), compared with ORs ranging from 0.32 to 0.52 in the other groups. Tubal ligation (yes vs no) was inversely associated with risk across all racial and ethnic groups, with the most pronounced association observed among Native Hawaiian/Pacific Islander participants (OR = 0.25; 95% CI, 0.13-0.48) compared with ORs of 0.68-0.78 in the other groups. There was no significant heterogeneity across racial and ethnic groups for age at menarche (*P* = .38 for heterogeneity) or breastfeeding (*P* = .56 for heterogeneity). Age at menarche (≥14 years compared with < 12 years) was associated with decreased EOC risk only among White participants (OR = 0.92; 95% CI, 0.85-0.99). Breastfeeding (yes vs no; parous women only) had an OR < 1 in all groups (Asian women, OR = 0.76 [95% CI, 0.57-0.99]; Native Hawaiian/Pacific Islander women, OR = 0.80 [95% CI, 0.41-1.55]; Hispanic women, OR = 0.96 [95% CI, 0.69-1.34]; and White women, OR = 0.75 [95% CI, 0.70-0.81]).

**Table 3:** Multivariable ORs^a^ (95% CI) for associations between exposures and epithelial ovarian cancer risk by racial and ethnic group

**Exposure**	**Asian** ^**b**^ **OR (95% CI)**	**Native Hawaiian/Pacific Islander** ^**b**^ **OR (95% CI)**	**Hispanic** **OR (95% CI)**	**White, non-Hispanic** **OR (95% CI)**	***P* for heterogeneity** ^**c**^
Age at menarche, y					
<12	1.00	1.00	1.00	1.00	.38
12-13	1.18 (0.90-1.55)	1.20 (0.65-2.21)	1.19 (0.87-1.62)	0.97 (0.91-1.05)	
≥14	1.26 (0.94-1.68)	1.29 (0.61-2.70)	1.02 (0.71-1.45)	0.92 (0.85-0.99)	
*P* for trend^d^	0.12	0.47	0.76	0.048	.15
OC use					
Never	1.00	1.00	1.00	1.00	.007
<5 y	0.55 (0.43-0.69)	0.30 (0.15-0.59)	0.86 (0.64-1.14)	0.75 (0.70-0.80)	
≥5 y	0.31 (0.22-0.42)	0.33 (0.14-0.79)	0.56 (0.40-0.79)	0.46 (0.43-0.50)	
*P* for trend^d^	<0.0001	0.003	0.002	<0.0001	.02
Parity					
0 live births	1.00	1.00	1.00	1.00	.002
1	0.56 (0.40-0.77)	0.40 (0.13-1.21)	0.81 (0.51-1.28)	0.71 (0.65-0.78)	
2	0.39 (0.29-0.51)	0.37 (0.14-0.96)	0.62 (0.41-0.93)	0.55 (0.51-0.60)	
≥3	0.32 (0.24-0.44)	0.10 (0.04-0.25)	0.52 (0.36-0.75)	0.50 (0.46-0.54)	
*P* for trend^d^	<0.0001	<0.0001	0.0002	<0.0001	.0001
Tubal ligation					
No	1.00	1.00	1.00	1.00	.006
Yes	0.68 (0.51-0.90)	0.25 (0.13-0.48)	0.70 (0.51-0.96)	0.78 (0.73-0.85)	
Breastfeeding^e^					
No	1.00	1.00	1.00	1.00	.56
Yes	0.76 (0.57-0.99)	0.80 (0.41-1.55)	0.96 (0.69-1.34)	0.75 (0.70-0.81)	
Postmenopausal hormone use^f^					
No	1.00	1.00	1.00	1.00	.62
Yes	0.82 (0.61-1.08)	0.60 (0.28-1.28)	0.97 (0.68-1.37)	0.91 (0.85-0.97)	
Endometriosis					
No	1.00	1.00	1.00	1.00	.95
Yes	1.59 (1.08-2.34)	1.37 (0.45-4.17)	1.51 (0.82-2.79)	1.42 (1.28-1.58)	
Hysterectomy^g^					
No	1.00	1.00	1.00	1.00	.58
Yes	1.30 (0.90-1.88)	1.06 (0.44-2.59)	1.42 (0.97-2.06)	1.12 (1.04-1.21)	
BMI, recent^h^					
<18.5	1.59 (0.90-2.80)		1.84 (0.38-9.00)	1.01 (0.82-1.25)	
18.5-24.9	1.00	1.00	1.00	1.00	.36
25.0-29.9	1.05 (0.80-1.39)	1.38 (0.69-2.76)	1.01 (0.73-1.39)	1.04 (0.97-1.12)	
30.0-34.9	1.77 (1.07-2.92)	0.98 (0.43-2.25)	1.24 (0.82-1.88)	1.14 (1.04-1.25)	
≥35.0	3.24 (1.35-7.82)	0.92 (0.43-1.96)	1.07 (0.69-1.67)	1.29 (1.16-1.25)	
*P* for trend^d^	0.03	0.81	0.52	<0.0001	.51
≥30.0 (vs 18.5-24.9)	2.05 (1.32-3.19)	0.95 (0.49-1.84)	1.16 (0.83-1.63)	1.20 (1.11-1.29)	.10
BMI, at age 18 y					
<18.5	1.21 (0.91-1.61)	1.86 (0.62-5.58)	1.00 (0.67-1.51)	0.91 (0.83-0.99)	
18.5-24.9	1.00	1.00	1.00	1.00	.33
25.0-29.9	1.18 (0.70-1.98)	0.73 (0.35-1.53)	1.22 (0.78-1.91)	1.24 (1.08-1.41)	
≥30.0	2.87 (0.85-9.67)	1.16 (0.48-2.80)	1.74 (0.79-3.82)	1.17 (0.92-1.48)	
*P* for trend^d^	0.83	0.47	0.18	<0.001	.44
Smoking					
Never	1.00	1.00	1.00	1.00	.001
Former	0.77 (0.65-1.26)	0.91 (0.48-1.73)	0.91 (0.65-1.26)	1.00 (0.94-1.06)	
Current	0.47 (0.30-0.74)	0.54 (0.26-1.15)	1.22 (0.76-1.96)	1.20 (1.10-1.31)	
Family history of breast cancer					
No	1.00	1.00	1.00	1.00	.15
Yes	1.01 (0.70-1.46)	0.71 (0.28-1.79)	1.80 (1.16-2.79)	1.24 (1.16-1.34)	
Family history of ovarian cancer					
No	1.00	1.00	1.00	1.00	.32
Yes	1.28 (0.65-2.52)		1.72 (0.96-3.08)	2.29 (1.99-2.64)	

Abbreviations: BMI, body mass index; OC, oral contraceptive; OR, odds ratio.

^a^ All models included study site, age group, and racial and ethnic group as strata variables; the exposure variable; and interaction terms for racial and ethnic group and the exposure variable. OC use (never [reference], < 5 y, ≥5 y) and parity (0 live births [reference], 1, 2, ≥3) were also included as adjustment variables.

^b^ Asian includes Chinese, Japanese, Korean, Filipino, Vietnamese, Thai. Native Hawaiian/Pacific Islander includes Hawaiian, Pacific Islander (Tongan, Samoan, Māori, Palauan, Chuukese, Micronesian).

^c^ *P* value for heterogeneity was calculated using the global Wald test on the interaction terms (race and ethnicity and categorical exposure variable(s), or race and ethnicity, and trend exposure variable).

^d^ *P* for trend was calculated using the median for that category: age at menarche (10, 12.5, 14 y); OC use (0, 2.5, 5 y); parity (0, 1, 2, 3 live births); recent BMI (18.5, 21.7, 27.5, 32.5, 35); BMI at age 18 y (18.5, 21.7, 27.5, 30.0).

^e^ Breastfeeding among parous women only.

^f^ Postmenopausal hormone use refers to use of estrogen only, estrogen plus progesterone, and unknown formulation types among postmenopausal women only.

^g^ Hysterectomy analysis excludes 1 study outlier (Mayo Clinic Ovarian Cancer Case-Control Study).

^h^ Recent BMI refers to 1 y before the reference date for all sites, except for Diseases of the Ovary and their Evaluation Study and Hawaii Ovarian Cancer Case-Control Study (5 years before reference date). BMI calculated as weight (kg) divided by height (m^2^).

Among factors that are typically associated with a higher risk of EOC, only smoking showed statistically significant heterogeneity across racial and ethnic groups (*P* = .001 for heterogeneity). Compared with never smokers, White current smokers had an increased risk of EOC (OR = 1.20; 95% CI, 1.10-1.31) and HGSC (OR = 1.24; 95% CI, 1.12-1.37; Table 4). In contrast, current smoking was inversely associated with risk among Asian participants (OR = 0.47; 95% CI, 0.30-0.74), with similar results when risk of HGSC was the outcome.

**Table 4:** Multivariable ORs^a^ (95% CI) for associations between exposures and high grade serous tubo-ovarian carcinoma^b^ risk by racial and ethnic group

**Exposure**	**Asian** ^**c**^ **OR (95% CI)**	**Native Hawaiian/Pacific Islander** ^**c**^ **OR (95% CI)**	**Hispanic** **OR (95% CI)**	**White, non-Hispanic** **OR (95% CI)**	***P* for heterogeneity** ^**d**^
Age at menarche, y					
<12	1.00	1.00	1.00	1.00	.48
12-13	1.25 (0.87-1.81)	1.85 (0.79-4.35)	1.11 (0.78-1.59)	1.01 (0.93-1.10)	
≥14	1.28 (0.86-1.89)	2.19 (0.82-5.82)	1.07 (0.71-1.60)	0.94 (0.85-1.03)	
*P* for trend^e^	0.23	0.10	0.69	0.23	.14
OC use					
Never	1.00	1.00	1.00	1.00	.08
<5 y	0.54 (0.39-0.74)	0.29 (0.12-0.73)	0.90 (0.65-1.27)	0.78 (0.72-0.84)	
≥5 y	0.35 (0.26-0.55)	0.44 (0.14-1.34)	0.55 (0.37-0.83)	0.48 (0.44-0.52)	
*P* for trend^e^	<0.0001	0.07	0.01	<0.0001	.25
Parity					
0 live births	1.00	1.00	1.00	1.00	.14
1	0.52 (0.34-0.82)	0.42 (0.09-1.95)	0.96 (0.56-1.66)	0.82 (0.73-0.91)	
2	0.42 (0.28-0.61)	0.44 (0.12-1.67)	0.80 (0.49-1.31)	0.69 (0.63-0.76)	
≥3	0.44 (0.30-0.64)	0.18 (0.05-0.58)	0.68 (0.44-1.05)	0.63 (0.58-0.69)	
*P* for trend^e^	<0.0001	<0.01	0.04	<0.0001	.05
Tubal ligation					
No	1.00	1.00	1.00	1.00	.06
Yes	0.76 (0.53-1.08)	0.28 (0.12-0.63)	0.84 (0.59-1.20)	0.85 (0.78-0.93)	
Breastfeeding^f^					
No	1.00	1.00	1.00	1.00	.55
Yes	0.78 (0.55-1.12)	0.59 (0.27-1.31)	0.98 (0.67-1.43)	0.75 (0.69-0.82)	
Postmenopausal hormone use^g^					
No	1.00	1.00	1.00	1.00	.60
Yes	0.84 (0.60-1.19)	0.65 (0.26-1.61)	0.97 (0.66-1.42)	1.01 (0.94-1.08)	
Endometriosis					
No	1.00	1.00	1.00	1.00	.38
Yes	1.84 (1.13-3.00)	1.07 (0.21-5.39)	1.23 (0.60-2.52)	1.17 (1.04-1.33)	
Hysterectomy^h^					
No	1.00	1.00	1.00	1.00	.98
Yes	1.21 (0.77-1.89)	1.27 (0.45-3.55)	1.30 (0.86-1.98)	1.81 (1.08-1.29)	
BMI, recent^i^					
<18.5	1.03 (0.46-2.29)		1.27 (0.13-12.8)	0.91 (0.71-1.17)	
18.5-24.9	1.00	1.00	1.00	1.00	.95
25.0-29.9	0.97 (0.68-1.38)	1.07 (0.44-2.56)	0.89 (0.61-1.27)	0.96 (0.89-1.04)	
30.0-34.9	1.42 (0.75-2.70)	0.87 (0.31-2.41)	1.02 (0.64-1.64)	1.02 (0.92-1.14)	
≥35.0	2.33 (0.79-6.81)	0.58 (0.21-1.64)	0.94 (0.57-1.55)	1.04 (0.92-1.18)	
*P* for trend^e^	0.26	0.35	0.85	0.60	.55
≥30.0 (vs 18.5-24.9)	1.60 (0.91-2.81)	0.71 (0.30-1.65)	0.98 (0.67-1.45)	1.03 (0.94-1.12)	.37
BMI, at age 18 y					
<18.5	1.13 (0.78-1.64)	1.73 (0.44-6.75)	0.89 (0.56-1.43)	0.96 (0.87-1.06)	
18.5-24.9	1.00	1.00	1.00	1.00	.39
25.0-29.9	0.66 (0.30-1.49)	0.54 (0.19-1.55)	0.97 (0.57-1.65)	1.20 (1.03-1.39)	
≥30.0	1.67 (0.35-7.92)	0.47 (0.10-2.24)	1.68 (0.66-4.24)	0.96 (0.87-1.06)	
*P* for trend^e^	0.39	0.09	0.45	0.09	.21
Smoking					
Never	1.00	1.00	1.00	1.00	.07
Former	0.83 (0.57-1.21)	1.16 (0.52-2.62)	1.07 (0.74-1.56)	1.03 (0.96-1.11)	
Current	0.45 (0.23-0.86)	0.68 (0.26-1.79)	1.30 (0.75-2.26)	1.24 (1.12-1.37)	
Family history of breast cancer					
No	1.00	1.00	1.00	1.00	.18
Yes	1.02 (0.69-1.68)	1.01 (0.31-3.37)	2.10 (1.30-3.40)	1.33 (1.22-1.45)	
Family history of ovarian cancer					
No	1.00	1.00	1.00	1.00	.45
Yes	1.61 (0.75-3.44)		2.03 (1.08-3.81)	2.76 (2.37-3.21)	

Abbreviations: BMI, body mass index; OC, oral contraceptive; OR, odds ratio.

^a^ All models included study site, age group, and racial and ethnic group as strata variables; the exposure variable; and interaction terms for racial and ethnic group and the exposure variable. OC use (never [reference], < 5 y, ≥ 5 y) and parity (0 [reference], 1, 2, ≥3 live births) were also included as adjustment variables.

^b^ High-grade serous tubo-ovarian carcinoma includes serous histology grades 2-4; serous histology with missing/unknown grade; endometrioid histology grade 3-4; and poorly differentiated epithelial histology grades 2-4.

^c^ Asian includes Chinese, Japanese, Korean, Filipino, Vietnamese, Thai. Native Hawaiian/Pacific Islander includes Hawaiian, Pacific Islander (Tongan, Samoan, Māori, Palauan, Chuukese, Micronesian).

^d^ *P* value for heterogeneity was calculated using the global Wald test on the interaction terms (race and ethnicity and categorical exposure variable(s), or race and ethnicity, and trend exposure variable).

^e^ *P* for trend calculated using the median for that category: age at menarche (10, 12.5, 14 y); OC use (0, 2.5, 5 y); parity (0, 1, 2, 3 live births); recent BMI (18.5, 21.7, 27.5, 32.5, 35); BMI at age 18 y (18.5, 21.7, 27.5, 30.0).

^f^ Breastfeeding among parous women only.

^g^ Postmenopausal hormone use refers to use of estrogen only, estrogen plus progesterone, and unknown formulation types among postmenopausal women only.

^h^ Hysterectomy analysis excludes 1 study outlier (Mayo Clinic Ovarian Cancer Case-Control Study).

^i^ Recent BMI refers to 1 y before the reference date for all sites, except for Diseases of the Ovary and their Evaluation Study and Hawaii Ovarian Cancer Case-Control Study (5 y before reference date). BMI calculated as weight (kg) divided by height (m^2^).

There was no significant heterogeneity across racial and ethnic groups for associations with hysterectomy, family history, or BMI. A significant increase in EOC risk for participants reporting a first-degree family history of breast cancer was only observed among Hispanic and White women (yes vs. no, OR = 1.80 [95% CI, 1.16-2.79]; and 1.24 [95% CI, 1.16-1.34], respectively; *P* = .15 for heterogeneity). Similarly, White participants with a reported family history of ovarian cancer had a significantly higher EOC risk (OR = 2.29; 95% CI, 1.99-2.64; *P* = .32 for heterogeneity), and a nonsignificant elevated risk was also observed for Hispanic participants (OR = 1.72; 95% CI, 0.96-3.08) and Asian participants (OR = 1.28; 95% CI, 0.65-2.52).

A positive association between recent BMI (≥30 compared with 18.5-24.9) and EOC risk was most pronounced in Asian women (OR = 2.05; 95% CI, 1.32-3.19), and an association was also observed in White women (OR = 1.20; 95% CI, 1.11-1.29; *P* = .10 for heterogeneity). Risk patterns with BMI at age 18 years were generally similar with recent BMI, but the findings were less pronounced.

We assessed whether there was heterogeneity in each of the risk factor associations for EOC overall between study sites and found between-study heterogeneity for recent BMI, smoking, OC use, parity, postmenopausal hormone use, and hysterectomy (*P* ≤ 0.01; Table S2). Despite this, the risk estimates were similar for both the fixed- and random-effects models of White participants and did not affect the study conclusions.

In analyses restricted to HGSC, none of the exposures had statistically significant heterogeneity for the risk associations between racial and ethnic groups (Table 4). Risk factor associations between racial and ethnic groups were generally similar for each exposure with risk of HGSC and EOC overall.

## Discussion

We conducted a comparative analysis of EOC risk factors across 4 racial and ethnic groups (Asian, Hispanic, Native Hawaiian/Pacific Islander, and White), using data from a large, pooled international consortium of 11 case-control studies. There was significant heterogeneity among racial and ethnic groups for associations with OC use, parity, tubal ligation, and smoking. The direction of the associations for most factors with risk of EOC (all histologic subtypes) was similar across racial and ethnic groups, with differences observed in the magnitude for some of the associations. This observation is consistent with the prior OCAC study.^9^ Smoking was not assessed in the prior OCAC study, and this was the only exposure for which we observed different directions in the association with risk between racial and ethnic groups. Importantly, in the present analysis, we evaluated additional exposures, and by separating Asian and Native Hawaiian/Pacific Islander participants, we were able to evaluate these groups in more detail.

We observed a more pronounced protective association with risk of EOC overall for both Asian and Native Hawaiian/Pacific Islander women with OC use (irrespective of the duration of use) and higher parity. Higher parity has a more pronounced inverse association with risk of endometrioid and clear cell carcinomas,^4^ both of which were more frequent in the Asian and Native Hawaiian/Pacific Islander groups. Similar findings were reported from the Multiethnic Cohort Study^10^ with respect to Japanese American participants showing the strongest inverse associations for ever use of OCs (≥5 years of use vs never use) and for parity (in comparisons of 3 or ≥ 4 children vs nulliparous) with risk of EOC overall. That study, however, did not report an association with OC use or parity for Native Hawaiian women, and it was limited by the small number of cases (*n* = 57). We observed that the inverse association for tubal ligation was most pronounced for Native Hawaiian/Pacific Islander women. As with parity, histotype-specific analyses have shown that the reduced risk for tubal ligation is strongest for clear cell carcinoma and could provide some explanation for this finding.^4^ Additionally, the prevalence of tubal ligation was considerably higher for Native Hawaiian/Pacific Islander control participants (42%) than Asian, Hispanic, and White control participants (range, 22%–26%). Although we adjusted for parity (both categorical and continuous), the association with tubal ligation could potentially reflect residual confounding. Results on tubal ligation were not reported in the earlier Multiethnic Cohort analysis^10^; therefore, it will be important to confirm the inverse association with tubal ligation for Native Hawaiian/Pacific Islander women in future studies.

We did not observe a significant interaction between recent BMI and EOC risk across racial and ethnic groups, but we noted that Asian women with a high BMI (>30) had a 2-fold increased risk of EOC compared with a 1.2-fold higher risk among White women and no association with BMI for Native Hawaiian/Pacific Islander or Hispanic participants. Although BMI was not associated with EOC risk among 161 Japanese American women in an earlier Multiethnic Cohort Study analysis, the group of Asian women included in the present analysis was larger and more diverse, with the 3 largest Asian groups represented by Japanese (*n* = 211 case and 324 control participants), Chinese (*n* = 249 case and 264 control participants), and Filipino women (*n* = 189 case and 215 control participants). Our finding could be related to the high cutpoints for BMI in this analysis, which may be less appropriate for Asian participants; however, it is broadly supported by observations of greater visceral adiposity and liver fat (adjusting for total fat mass) among Japanese American compared with White women in the Multiethnic Cohort Study.^19^ A better understanding of different fat components by racial and ethnic groups is needed.

Current vs never smoking was associated with an unexpected lower risk of EOC and HGSC in Asian participants. This finding contrasts with the higher risk with current smoking observed for White participants and no association for Native Hawaiian/Pacific Islander and Hispanic participants in our study. In histological subtype-specific analyses focused on mostly White participants, a prior OCAC study found an approximate 1.3-fold increased risk for mucinous EOC in current vs never smokers.^20^ Similar findings were reported for ever vs never smoking in a prospective Ovarian Cancer Cohort Consortium study. In analysis focusing on clear cell EOC, an inverse association was observed for both current and former smoking vs never smoking, with risk of clear cell EOC in an OCAC analysis.^20^ In the Ovarian Cancer Cohort Consortium study, there was no association for ever vs never smoking, but there was a 32% lower risk of clear cell EOC per 20 pack-years.^4^ In Asian populations, the incidence of clear cell EOC has been reported to be higher,^21^ which could account for the combined EOC result in the present study. However, this would not explain the similar inverse associations with smoking for Asian participants in analyses of HGSC alone. An association between ever vs never smoking with EOC risk was not observed in the Multiethnic Cohort Study in Japanese American or in other racial and ethnic groups.^10^ It is possible that recall bias in cases compared with control participants could have been a factor in this result, and it requires validation in additional study populations.

The main strengths of this study are the detailed exposure assessments with extensive information on EOC risk factors and the large number of EOC cases, ensuring racial and ethnic diversity in the study population. Importantly, we expanded on the earlier OCAC study^9^ to report separately on EOC risk factor associations for Asian and Native Hawaiian/Pacific Islander women; these have only been reported in 1 previous study, to our knowledge.^10^ A limitation is the use of data from case-control studies, potentially subject to selection bias, and retrospective ascertainment of exposures and self-report, which may be subject to recall bias. However, case-control studies are required to efficiently accrue a substantial number of cases, particularly to study EOC risk factors for underrepresented racial and ethnic groups. Despite the large size of this study, it was not possible to tease out whether some of the differences in risk factor associations between racial and ethnic groups could be explained by histological subtype, and the lack of heterogeneity in risk factor associations across racial and ethnic groups in analysis of HGSC alone may support this. Future analyses including population-attributable risk calculations^13^^,^^22^ may be a suitable way to account for differences in the prevalence of exposures across racial and ethnic groups. Our study focused on EOC risk factor associations for Asian, Hispanic, Native Hawaiian/Pacific Islander, and White participants, and these data were sourced from studies representing populations in high-income countries (United States, Canada, and Australia); thus, the results may not be generalizable to the racial and ethnic populations outside these countries, due to potential differences in dietary patterns or contraceptive practices. Additionally, we were unable to differentiate between subpopulations of Asian participants, nor those born in Asia vs elsewhere. Finally, we analyzed multiple exposures and cannot discount the possibility of chance findings.

In summary, to our knowledge, this study was the first large-scale consortium analysis to evaluate EOC risk factor associations separately for Asian and Native Hawaiian/Pacific Islander populations. There were notable differences in risk associations for EOC across racial and ethnic groups, including a more pronounced protective association for tubal ligation in Native Hawaiian/Pacific Islander women. There were also strong inverse associations for parity and OC use with risk of EOC for both Native Hawaiian/Pacific Islander and Asian women. Together, these findings reinforce the importance of greater inclusion of racially and ethnically diverse populations in epidemiologic studies to improve strategies for the primary prevention of ovarian cancer.

## Supplementary material

Supplementary material is available at *American Journal of Epidemiology* online.

## Supplementary Material

Web_Material_kwae076

## Data Availability

The data used for this analysis will be shared upon approval of a data request by the Ovarian Cancer Association Consortium (https://ocac.ccge.medschl.cam.ac.uk/) Data Access Coordinating Committee and participating studies with appropriate human participants’ approval and data transfer agreements.
