# Supplementary material for: Racial and ethnic differences in epithelial ovarian cancer risk: an analysis from the Ovarian Cancer Association Consortium
Source: Am J Epidemiol. 2024 May 21;193(9):1242–52. doi: 10.1093/aje/kwae076 (PMC11369223; doi:10.1093/aje/kwae076)
Supplement: Web_Material_kwae076 [file web_material_kwae076.docx]

**Supplementary material**

**Title**: Racial and ethnic differences in epithelial ovarian cancer risk: an analysis from the Ovarian Cancer Association Consortium

**Authors**: Nicola S. Meagher,* Kami K. White,* Lynne R. Wilkens, Elisa V. Bandera, Andrew Berchuck, Michael E. Carney, Daniel W. Cramer, Kara L. Cushing-Haugen, Susan Jordan, Scott H. Kaufmann, Nhu D. Le, Malcolm C. Pike, Marjorie Riggan, Bo Qin, Joseph H. Rothstein, Linda Titus, Stacey J. Winham, Hoda Anton-Culver, Jennifer A. Doherty, Ellen L. Goode, Celeste Leigh Pearce, Harvey A. Risch, Penelope M. Webb on behalf of the AOCS Group, Linda S. Cook, Marc T. Goodman, Holly R. Harris, Loic Le Marchand, Valerie McGuire, Paul D.P. Pharoah, Danja Sarink, Joellen M. Schildkraut, Weiva Sieh, Kathryn L. Terry, Pamela J. Thompson, Alice S. Whittemore, Anna H. Wu, Lauren C. Peres, Melissa A. Merritt

*These authors contributed equally to this work

**Table of contents:**

**Page Table**

**2 Table S1**: Age-standardized participant characteristics by racial and ethnic group

**4 Table S2**: Multivariable Odds Ratios (95% CI) for associations between exposures and epithelial ovarian cancer risk for Non-Hispanic White participants only with assessment of study heterogeneity.

| **Table S1**: Age-standardized^a^ participant characteristics by racial and ethnic group | | | | | | | | | |
| --- | --- | --- | --- | --- | --- | --- | --- | --- | --- |
|  | Asian^b^ | | Native Hawaiian/Pacific Islander^b^ | | | Hispanic | | White, Non-Hispanic |  |
|  | Controls (%) | | Controls (%) | | | Controls (%) | | Controls (%) |  |
| Age at menarche (years) |  | |  | | |  | |  |  |
| <12 | 21.5 | | 35.9 | | | 24.8 | | 19.4 |  |
| 12-13 | 46.2 | | 43.9 | | | 47.3 | | 55.0 |  |
| ≥14 | 32.4 | | 20.2 | | | 27.9 | | 25.6 |  |
| OC use |  | |  | | |  | |  |  |
| Never | 51.3 | | 41.4 | | | 46.8 | | 26.5 |  |
| <5 years | 30.9 | | 42.8 | | | 31.4 | | 32.8 |  |
| ≥5 years | 17.8 | | 15.9 | | | 21.8 | | 40.7 |  |
| Parity |  | |  | | |  | |  |  |
| 0 live births | 16.1 | | 6.8 | | | 13.3 | | 17.4 |  |
| 1 | 14.9 | | 9.3 | | | 11.5 | | 12.9 |  |
| 2 | 31.6 | | 15.5 | | | 24.3 | | 34.7 |  |
| ≥3 | 37.4 | | 68.4 | | | 51.4 | | 34.9 |  |
| Tubal ligation |  | |  | | |  | |  |  |
| No | 76.6 | | 52.7 | | | 75.5 | | 76.2 |  |
| Yes | 23.4 | | 47.3 | | | 24.5 | | 23.8 |  |
| Breastfeeding^c^ |  | |  | | |  | |  |  |
| No | 21.4 | | 28.4 | | | 34.0 | | 30.1 |  |
| Yes | 78.6 | | 71.6 | | | 66.0 | | 69.9 |  |
| Menopausal status |  | |  | | |  | |  |  |
| Pre/peri | 36.5 | | 29.4 | | | 32.2 | | 33.4 |  |
| Post | 63.5 | | 70.6 | | | 67.8 | | 66.6 |  |
| Postmenopausal hormone use^d^ | |  | |  |  | |  | |  |
| No | | 53.3 | | 65.3 | 61.4 | | 48.5 | |  |
| Yes | | 46.7 | | 34.5 | 38.4 | | 51.6 | |  |
| Endometriosis | |  | |  |  | |  | |  |
| No | | 92.2 | | 94.7 | 96.0 | | 92.3 | |  |
| Yes | | 7.8 | | 5.3 | 4.0 | | 7.7 | |  |
| Hysterectomy | |  | |  |  | |  | |  |
| No | | 91.2 | | 88.4 | 86.2 | | 83.4 | |  |
| Yes | | 8.8 | | 11.6 | 13.8 | | 16.6 | |  |
| BMI, recent^e^ | |  | |  |  | |  | |  |
| <18.5 kg/m^2^ | | 3.5 | | 0.4 | 0.4 | | 2.1 | |  |
| 18.5-24.9 | | 65.0 | | 29.4 | 38.6 | | 48.8 | |  |
| 25.0-29.9 | | 24.8 | | 27.8 | 34.7 | | 28.8 | |  |
| 30.0-34.9 | | 5.5 | | 17.9 | 14.3 | | 12.5 | |  |
| ≥35.0 | | 1.0 | | 24.4 | 12.0 | | 7.8 | |  |

|  | Asian^b^ | Native Hawaiian/Pacific Islander^b^ | Hispanic | White, Non-Hispanic |
| --- | --- | --- | --- | --- |
| BMI, age 18 years | Controls (%) | Controls (%) | Controls (%) | Controls (%) |
| <18.5 kg/m^2^ | 19.4 | 5.7 | 14.7 | 17.9 |
| 18.5-24.9 | 74.3 | 65.8 | 74.6 | 74.2 |
| 25.0-29.9 | 5.6 | 19.4 | 8.8 | 6.1 |
| ≥30.0 | 0.7 | 9.1 | 1.9 | 1.8 |
| Smoking |  |  |  |  |
| Never | 74.3 | 50.5 | 61.6 | 53.3 |
| Former | 16.5 | 25.8 | 29.2 | 35.7 |
| Current | 9.3 | 23.7 | 9.3 | 11.0 |
| Family history of breast cancer |  |  |  |  |
| No | 81.3 | 54.9 | 89.1 | 83.4 |
| Yes | 18.7 | 45.1 | 10.9 | 16.6 |
| Family history of ovarian cancer |  |  |  |  |
| No | 95.0 | 96.4 | 93.7 | 97.0 |
| Yes | 5.0 | 3.6 | 6.3 | 3.0 |

Abbreviations: BMI (Body Mass Index); OC (Oral Contraceptive).

The following variables were missing for certain study sites: breastfeeding (MAY); endometriosis (OVA, STA); BMI recent (OVA, STA); BMI at age 18 (MAY, OVA, STA); postmenopausal hormone use (STA). One study (MAY) was an outlier and excluded from hysterectomy analysis.

^a^Values were standardized to the age distribution of the study population in 10-year age groups.

^b^Asian includes Chinese, Japanese, Korean, Filipino, Vietnamese, Thai. Native Hawaiian/Pacific Islander includes Hawaiian, Pacific Islander (Tongan, Samoan, Maori, Palauan, Chuukese, Micronesian).

^c^Breastfeeding refers to parous women only.

^d^Postmenopausal hormone use refers to use of estrogen only, estrogen plus progesterone and unknown formulation types among postmenopausal women only.

^e^Recent BMI refers to 1 year before the reference date (date of diagnosis for cases, date of interview for controls) for all sites, except for DOV and HAW (5 years before the reference date).

**Table S2**. Multivariable Odds Ratios^a^ (95% CI) for associations between exposures and epithelial ovarian cancer risk for Non-Hispanic White participants only with assessment of study heterogeneity.

|  | Fixed Effects Model | Random Effects Model |  |  | |
| --- | --- | --- | --- | --- | --- |
| Exposure | OR (95% CI) | OR (95% CI) | Q | p for heterogeneity | |
| Age at menarche |  |  |  |  | |
| <12 years | 1.00 | 1.00 |  |  | |
| 12-13 years | 0.97 (0.91, 1.04) | 0.97 (0.89, 1.06) | 14.0 | 0.17 | |
| ≥14 years | 0.92 (0.85, 1.00) | 0.92 (0.84, 1.01) | 11.2 | 0.35 | |
| p for trend^b^ | 0.98 (0.96, 1.00) | 0.98 (0.96, 1.00) | 12.5 | 0.25 | |
| OC use |  |  |  |  | |
| Never | 1.00 | 1.00 |  |  | |
| <5y | 0.75 (0.70, 0.81) | 0.75 (0.67, 0.84) | 23.4 | 0.01 | |
| 5y+ | 0.46 (0.43, 0.49) | 0.46 (0.42, 0.51) | 17.0 | 0.07 | |
| p for trend^b^ | 0.86 (0.84, 0.87) | 0.86 (0.84, 0.88) | 17.9 | 0.06 | |
| Parity |  |  |  |  | |
| 0 live births | 1.00 | 1.00 |  |  | |
| 1 | 0.71 (0.64, 0.78) | 0.70 (0.62, 0.79) | 15.0 | 0.13 | |
| 2 | 0.55 (0.51, 0.60) | 0.53 (0.45, 0.63) | 42.4 | <0.0001 | |
| 3+ | 0.50 (0.46, 0.54) | 0.48 (0.40, 0.58) | 47.6 | <0.0001 | |
| p for trend^b^ | 0.79 (0.77, 0.81) | 0.78 (0.74, 0.83) | 52.8 | <0.0001 | |
| Tubal ligation |  |  |  |  | |
| No | 1.00 | 1.00 |  |  | |
| Yes | 0.78 (0.72, 0.83) | 0.78 (0.72, 0.83) | 3.6 | 0.97 | |
| Breastfeeding^c^ |  |  |  |  | |
| No | 1.00 | 1.00 |  |  | |
| Yes | 0.75 (0.69, 0.80) | 0.75 (0.69, 0.83) | 13.2 | 0.15 | |
| Postmenopausal hormone use^d^ |  |  |  |  | |
| No | 1.00 | 1.00 |  |  | |
| Yes | 0.91 (0.85, 0.97) | 0.91 (0.84, 0.98) | 12.0 | <0.01 |  |
| Endometriosis |  |  |  |  | |
| No | 1.00 | 1.00 |  |  | |
| Yes | 1.42 (1.28, 1.57) | 1.42 (1.28, 1.57) | 6.0 | 0.65 | |
| Hysterectomy^e^ |  |  |  |  | |
| No | 1.00 | 1.00 |  |  | |
| Yes | 1.12 (1.04, 1.21) | 1.12 (0.99, 1.28) | 19.7 | 0.01 | |
| BMI, recent^f^ |  |  |  |  | |
| ln(BMI) | 1.42 (1.23, 1.64) | 1.28 (0.97, 1.69) | 26.7 | <0.01 | |
| BMI, at age 18 |  |  |  |  | |
| <18.5 kg/m^2^ | 0.91 (0.83, 0.99) | 0.91 (0.83, 0.99) | 6.3 | 0.50 | |
| 18.5-24.9 | 1.00 | 1.00 |  |  | |
| 25.0-29.9 | 1.24 (1.09, 1.41) | 1.24 (1.09, 1.41) | 5.6 | 0.58 | |
| 30.0+ | 1.16 (0.92, 1.47) | 1.18 (0.91, 1.51) | 7.6 | 0.37 | |
|  | Fixed Effects Model | Random Effects Model |  |  | |
| Exposure | OR (95% CI) | OR (95% CI) | Q | p for heterogeneity | |
| Smoking |  |  |  |  |  |
| Never | 1.00 | 1.00 |  |  |  |
| Former | 1.00 (0.94, 1.06) | 1.00 (0.94, 1.06) | 5.9 | 0.83 |  |
| Current | 1.20 (1.10, 1.32) | 1.10 (0.91, 1.34) | 40.6 | <0.0001 |  |
| Family history of breast cancer |  |  |  |  |  |
| No | 1.00 | 1.00 |  |  |  |
| Yes | 1.24 (1.15, 1.34) | 1.24 (1.15, 1.34) | 6.4 | 0.78 |  |
| Family history of ovarian cancer |  |  |  |  |  |
| No | 1.00 | 1.00 |  |  |  |
| Yes | 2.29 (1.98, 2.63) | 2.29 (1.98, 2.63) | 5.1 | 0.89 |  |

Abbreviations: BMI (body mass index); CI (confidence interval); OR (odds ratio)

^a^ All models included study site and age group as strata variables, the exposure variable, and interaction term(s) for study site and the exposure variable. OC use (never (Ref), <5 years, ≥ 5 years) and parity (0 live births (Ref), 1, 2, 3+) were also included as adjustment variables.

^b^ p for trend calculated using the median for that category: age at menarche (10, 12.5, 14); OC use (0, 2.5, 5); parity (0 live births, 1, 2, 3)

^c^ Breastfeeding: among parous women only.

^d^ Postmenopausal hormone use refers to use of estrogen only, estrogen plus progesterone and unknown formulation types among postmenopausal women only.

^e^ Hysterectomy analysis excludes one study outlier (MAY).

^f^ Recent BMI refers to 1 year before the reference date for all sites, except for DOV and HAW (5 years before reference date).
